# Noise Induced Hearing Loss and Tinnitus—New Research Developments and Remaining Gaps in Disease Assessment, Treatment, and Prevention

**DOI:** 10.3390/brainsci10100732

**Published:** 2020-10-13

**Authors:** Tang-Chuan Wang, Ta-Yuan Chang, Richard Tyler, Ying-Ju Lin, Wen-Miin Liang, Yio-Wha Shau, Wei-Yong Lin, Yi-Wen Chen, Chia-Der Lin, Ming-Hsui Tsai

**Affiliations:** 1 Department of Public Health, China Medical University, Taichung City 40402, Taiwan; 2 School of Medicine, China Medical University, Taichung City 40402, Taiwan; d6355@mail.cmuh.org.tw (C.-D.L.); minghsui5121@gmail.com (M.-H.T.); 3 Department of Otolaryngology-Head and Neck Surgery, China Medical University Hsinchu Hospital, Zhubei City, Hsinchu County 302056, Taiwan; 4 Department of Occupational Safety and Health, China Medical University, Taichung City 40402, Taiwan; tychang@mail.cmu.edu.tw; 5 Department of Otolaryngology-Head and Neck Surgery, University of Iowa, Iowa City, IA 52242-1396, USA; rich-tyler@uiowa.edu; 6 School of Chinese Medicine, China Medical University, Taichung City 40402, Taiwan; yjlin.kath@gmail.com; 7 Department of Health Services Administration, China Medical University, Taichung City 40402, Taiwan; wmliang@mail.cmu.edu.tw; 8 College of Biomedical Engineering, China Medical University, Taichung City 40402, Taiwan; ywshau@mail.cmu.edu.tw; 9 Graduate Institute of Integrated Medicine, China Medical University, Taichung City 40402, Taiwan; linwy@mail.cmu.edu.tw; 10 Graduate Institute of Biomedical Sciences, China Medical University, Taichung City 40402, Taiwan; evinchen@gmail.com

**Keywords:** central auditory system, cochlear damage, deafferentation, hidden hearing loss, maladaptive plasticity, neural plasticity, noise induced hearing loss, tinnitus

## Abstract

Long-term noise exposure often results in noise induced hearing loss (NIHL). Tinnitus, the generation of phantom sounds, can also result from noise exposure, although understanding of its underlying mechanisms are limited. Recent studies, however, are shedding light on the neural processes involved in NIHL and tinnitus, leading to potential new and innovative treatments. This review focuses on the assessment of NIHL, available treatments, and development of new pharmacologic and non-pharmacologic treatments based on recent studies of central auditory plasticity and adaptive changes in hearing. We discuss the mechanisms and maladaptive plasticity of NIHL, neuronal aspects of tinnitus triggers, and mechanisms such as tinnitus-associated neural changes at the cochlear nucleus underlying the generation of tinnitus after noise-induced deafferentation. We include observations from recent studies, including our own studies on associated risks and emerging treatments for tinnitus. Increasing knowledge of neural plasticity and adaptive changes in the central auditory system suggest that NIHL is preventable and transient abnormalities may be reversable, although ongoing research in assessment and early detection of hearing difficulties is still urgently needed. Since no treatment can yet reverse noise-related damage completely, preventative strategies and increased awareness of hearing health are essential.

## 1. Introduction

Long-term noise exposure can result in sensorineural deafness commonly called noise induced hearing loss (NIHL). The phantom auditory sensation, tinnitus, can also result from noise exposure, although understanding of the underlying pathophysiology of tinnitus has been limited to date. According to the World Health Organization, in 2020 about 466 million individuals worldwide have disabling hearing loss, which is expected to almost double by 2050; about 34 million children have hearing loss; and up to 60% of cases are preventable [1]. Among teenagers and young adults worldwide, about 1.1 billion have NIHL, mainly from the use of headphones and personal music players or other recreational noise [2]. Exposure to excessive occupational noise (sound intensity over 85 dB A) is a known cause of NIHL in adults, with a prevalence of 12%–19% among US workers [3,4], 15%–34% in Canada [5], and 10% in Japan [6]. Similarly, a systematic review found that occupational noise exposure causes 7%–21% of NIHL among workers, with the lowest rates in the industrialized countries and highest in the developing countries [7]. The industries and activities most associated with higher risks of NIHL have been identified as military, forestry, agriculture, fishery, and hunting [4,7]. Some studies conducted in Taiwan have addressed NIHL in workers who were exposed to both organic solvents and noise in specific industry sectors, such as oil refineries, liquid petroleum gas infusion factories, and adhesive materials manufacturing [8,9,10]. Workers exposed to both of these hazards were found to significantly more likely to have hearing loss of ≥25 dB than those in the noise-only group and/or administrative clerks [8,9]. Self-reported hearing-loss symptoms in oil refinery workers were found to correlate with increased hearing loss at both low and high frequencies, suggesting that self-reported symptoms provide early signs of NIHL [10]. Furthermore, the use of handheld power and pneumatic tools from chain saws to dentistry equipment that expose workers to both noise and vibration in the upper body are considered hazardous to hearing [11,12,13]. Workers in most countries must comply with workplace regulations regarding noise exposure. Workers involved in noisy professions are required to wear protective gear, and employers may be mandated to limit workers’ hours to reduce noise exposure, submit regular noise monitoring of the working environment, and even provide workers with health check-ups [14,15,16,17].

Permanent hearing loss may develop as a result of noise exceeding 89 dB A for more than five hours a week [18]. Other factors that contribute to the acceleration of NIHL and related tinnitus might include smoking, diabetes, and low levels of exercise, as well as non-modifiable factors such as aging, genetics and racial/ethnic influences. Although gender is not considered to be a major risk factor, among young people, males are found to be more likely than females to engage in risk-related behavior such as high-risk noise activities [19]. However, a study conducted in China found that age and gender were most strongly associated with hearing loss in older adults, and other prominent risk factors in this population were ear disease, hypertension, atherosclerosis, noise exposure and ototoxic drugs [20]. Head and neck injuries can also trigger the mechanisms that result in tinnitus when abnormal somatosensory input affects auditory pathways [21]. Several studies have highlighted the involvement of non-auditory brain regions in the pathophysiology of tinnitus, including the loss of connection with the limbic system that normally “tunes out” tinnitus signals originating from auditory pathways, and the dysfunctional network of auditory-sensory and fronto-striatal circuits [22,23,24]. Yet many questions remain to be answered.

This review explores questions about the early signs of hearing difficulties, links between tinnitus and noise exposure, the preventability of NIHL, possible reversal of sensorineural hearing damage, detection of so-called ‘hidden’ hearing loss, developments in assessment and detection, appropriate prevention plans, adaptation of the central auditory system to changes, and treatments in development.

## 2. Overview of Current Knowledge

Extended exposure to noise can result in continuous apoptosis of hair cells and degeneration of spiral ganglion neurons, gradually decreasing speech recognition and increasing hearing thresholds, which can lead to permanent hearing loss [25]. At the same time, noise exposure causes cochlear blood vessels to contract and disturbs cell energy metabolism, producing huge amounts of free radicals such as reactive oxygen species (ROS). One treatment goal could be to alleviate apoptosis and cochlear damage before permanent hearing loss occurs.

Audiograms confirming hearing loss have long been recognized as the most important condition predisposing individuals to tinnitus [26,27]. Study results have suggested that tinnitus is generated by aberrant neural changes in the central auditory structures that occur when these structures are deafferented by damage to the cochlea, as detected with audiograms or other more-sensitive measures, such as imaging, magnetoencephalography, and auditory evoked potential [28,29,30,31]. Neural plasticity is involved in these changes, including spontaneous activity, bursting and synchronous activity among neurons in subcortical and cortical auditor regions, and strengthened inputs from somatosensory to deafferented auditory structures [32]. Individuals who have developed severe deafness after long-term NIHL can only rely on cochlear implants or hearing aids to improve hearing; regeneration of hair cells would be needed in order to restore hearing completely [18]. Maladaptive plasticity in brain regions might underlie tinnitus-associated neural changes, causing increases in spontaneous firing rates and synchrony between neurons in the auditory cortex and other brain regions [21]. The Psychological Model of Tyler et al (1992) suggested that the neural activity associated with tinnitus should be separated and distinguished from the neural activity related to reactions to the tinnitus [33]. However, while several different approaches are suggested to alleviate reactions to tinnitus, no cure is known.

Animal models have contributed important information to the study of NIHL. In animals hearing loss after exposures of only 24 h can damage sensory hair cells in the inner ear [34]. Tinnitus-associated neural changes suggested by animal models indicate changes in the cochlear nucleus and extend to the auditory cortex and other regions of the brain [34]. In human studies, functional imaging of patients with tinnitus has also shown that neural changes are observed in nonauditory brain regions involved in attention, emotion, and memory [24], consistent with the Psychological Model proposed by Tyler et al. (1992). Emotional and attention-related states are suggested to contribute to maintaining tinnitus [21]. While most tinnitus results from NIHL and is accompanied by changes in central auditory pathways, the location of these changes is unclear and controversial. Imaging studies (e.g., functional magnetic resonance imaging [fMRI], positron emission tomography, magnetoencephalography) of tinnitus patients have demonstrated activity in non-auditory, limbic brain structures, including the hippocampus and amygdala [35]. Although the initial generation of the tinnitus signal likely arises in the auditory system, the reactions must involve the limbic system and perhaps other brain areas [23].

Several recent review studies have emphasized the maladaptive plasticity of NIHL, covering detailed neuronal aspects of triggers of tinnitus, mechanisms underlying generation of tinnitus in the cochlear nucleus after noise-induced deafferentation, likely routes of transmission of tinnitus signals along the ascending nuclei, as well as treatment approaches to reduce the impact of tinnitus on the quality of life of affected individuals [21,36,37,38,39]. A general consensus is that NIHL is preventable and that there is much more to learn about tinnitus and its underlying mechanisms and undisputed connection with NIHL. All authors emphasize the importance of increasing public awareness of noise pollution and potential hearing loss in today’s world.

## 3. Hearing Loss Assessment

Most current audiological assessments are noninvasive and focus on peripherally presented sounds, measuring the individual’s ability to hear different frequencies. Pure-tone audiometry identifies an individual’s hearing threshold and can determine the degree, type and configuration of hearing loss as a basis for diagnosis and subsequent management. It is used effectively for threshold audiometry and screening, with 92% sensitivity and 94% specificity in detection of sensorineural hearing impairment [40]. More comprehensive assessment may also involve complete otolaryngologic examination, particularly if the patient’s complaints include tinnitus, vertigo, otalgia or otorrhea, in addition to perceived hearing loss.

Retro-cochlear screening is typically performed when hearing loss is sudden or progresses rapidly, or is an asymmetric sensorineural loss. This can involve auditory brainstem response testing and/or gadolinium-enhanced magnetic resonance imaging (MRI) to detect retro-cochlear pathology such as vestibular schwannomas, cerebellopontine angle tumors, multiple sclerosis or stroke, among other possibilities [41].

Structural neuroimaging studies are increasingly being performed to evaluate the effects of hearing loss on the brain. MRI technology, in particular, is sensitive to the changes in white and gray matter structures and allows quantitative evaluation at the macroscopic level. Structural MRI can be combined with fMRI to provide brain mapping and biomarkers for tracking amplification longitudinally [42]. Diffusion tensor imaging of the auditory pathway and of the auditory nerve may be performed in patients with long-term unilateral hearing loss [43,44].

More sophisticated laboratory studies are also being conducted to clarify central nervous system (CNS) involvement in hearing loss. Because auditory processing begins in the cochlea and the sounds detected by sensory hair cells are then transmitted to the CNS by multiple types of spiral ganglion neurons, hearing assessment sometimes targets the post-cochlear regions, including the CNS. The mechanically sensitive hair cells in the cochlea relay auditory information to the CNS by releasing glutamate to the spiral ganglion neurons (SGN). Different SGN subtypes have been identified in molecular studies and found to have different morphology, innervation patterns and firing characteristics [45]. Electrophysiological features of the SGN have been distinguished tonotopically in the same cochlear regions in various animal studies, showing different levels of susceptibility to aging and neurotoxicity associated with noise [46]. Single-fiber recording studies in aging and noise-exposed rats have indicated that high-threshold neurons are reduced in number, but the precise mechanisms are still poorly understood. Ongoing genetic studies and single-cell RNA analysis of spiral ganglion neurons are underway to assess CNS involvement in hearing loss.

Current difficulties in clinical audiological assessment include the fact that some individuals with hearing thresholds within normal limits (= < 25 dB HL) still find it difficult to hear clearly in noisy environments. This condition is often referred to as “hidden hearing loss,” which differs distinctly from loss due to shifts in the hearing threshold. However, it should be appreciated that the 0 dB HL is the average hearing of young adults. Some people had thresholds of −10 dB HL when they were 21 years old. If their thresholds are 0 dB HL at the present, they now have a sensorineural hearing loss. Individuals who self-report tinnitus can also can have audiograms with thresholds of 25 dB HL or less. Tinnitus can be measured, focusing on its pitch, loudness and mask ability and using a variety of magnitude estimation procedures [47,48]. The individual’s reaction to the tinnitus, such as thoughts, emotions, hearing, sleep and concentration, can also be measured using questionnaires [49,50,51]. For example, the loudness of tinnitus can be measured by increasing the level of a pulsed tone of 500 Hz in 2 dB steps, until the sound reaches the intensity perceived by the patient as equal to his/her tinnitus loudness [51]. Hearing loss, which may occur after exposure to even a single loud noise, is thought to damage the synapses between cochlear hair cells and auditory nerve fibers, but is not always detected by pure tone threshold. In mice, there have been approaches to measuring the ability of neurons in the auditory midbrain to response to soft and loud sound environments [52].

Gap-prepulse inhibition of the acoustic startle reflex (GPIAS) is reserved for tinnitus evaluation, particularly in animal models of tinnitus [53]. The testing method is predicated on the idea that tinnitus fills in this gap during the testing process. The GPIAS test method has been used in animal models to assess tinnitus and is now being tested in humans to indicate the presence of tinnitus [53]. However, this approach is still controversial and its value in assessment remains to be demonstrated.

An overall goal of assessment is to detect early hearing problems in a timely manner so that steps can be taken to prevent further damage. Auditory assessment, therefore, must consider the early transient changes, including when and how various perceptual abnormalities begin to emerge—and, importantly, to do this before permanent hearing impairment occurs.

## 4. Current Treatment Methods

Treatments are necessarily different when targeting hearing loss or tinnitus alone. Stem cell therapy, for example, targets mainly hearing loss, and stimulation of the vagus nerve targets mainly tinnitus. Here we review current treatment methods, including those commonly used for treatment of hearing loss and a separate subsection for “tinnitus management.”

### 4.1. Cochlear Implants

Although hearing aids have been the conventional solution to hearing loss associated with aging or noise exposure, surgically implanted electronic devices may be indicated for adults and children with profound sensorineural hearing loss. Although these cochlear implants have been used for thirty years, and usage increases steadily, improvement cannot be predicted easily. Speech perception can present challenges among recipients [54]. Poor outcomes cannot always be explained, although successful outcomes are predominant. Today, cochlear implant hearing performance can be measured in situ, which is a relatively new research method and expected to improve the success rate of cochlear implants [54,55].

### 4.2. Pharmacologic Therapy

Pharmacologic agents applied in the treatment of hearing loss include glutamate inhibitors such as caroverine (Tinnex), derivatives of trimetazidine anti-ischemic agent such as trimetazidine dihydrochloride (Adexor), and lidocaine perfusion of the inner ear specifically for tinnitus. These treatments are not available in all countries and reports of effectiveness are controversial [56,57,58].

The damage in NIHL involves mechanical shearing force, oxidative damage and glutamate excitotoxicity, any of which could be eventual targets for post-exposure treatment and prevention. However, avoiding noise remains the present reality in terms of agents of prevention.

### 4.3. Antioxidants

Animal studies show that antioxidant enzymes from the body’s biochemical pathways, especially glutathione, increase in the cochlea after noise exposure. Acting as an effective antioxidant, glutathione scavenges free radicals in the cochlea after noise exposure, and transcription of large quantities of antioxidant enzymes SOD1 and HO-1 genes also provides a certain protective effect [59]. These observations in animal models suggest that effects of intense noise, whether sudden (as in gunshot) or over time (as in workplace noise), may be reduced or alleviated through supplementation of exogenous antioxidants.

### 4.4. Tinnitus Management

Since no cure is available for tinnitus, management usually requires a broad, comprehensive approach to therapy. Treatment typically includes counseling, hearing aids, and sound therapy [60]. A variety of counseling strategies exist, from directive to collaborative [48,61]. The primary areas affected are thoughts and emotions, hearing, concentration, and sleep [51]. Meanwhile, Cognitive Behavior Therapy has been both emphasized and challenged [48,62]. Hearing aids can help with communication and tinnitus [63]. A variety of sound therapy approaches are available (35), and a recent study documents the effectiveness of sound therapy for some individuals [64]. Acupuncture and electromagnetic stimulation have also been attempted. Dietary supplements are used extensively in the treatment of tinnitus, including gingko biloba, lipo-flavonoids, manganese, magnesium, melatonin, vitamin B12, and zinc [65,66].

## 5. Treatments in Development

Results of recent clinical trials on hearing loss evaluation, prevention and treatment are pointing the way beyond current practices and available screening and treatment strategies. The challenges are many, including the difficulties in choosing the subtypes of tinnitus and of focusing on individual differences in hearing loss [48,67]. Here we describe emerging treatments for hearing loss (5.1, 5.2), especially tinnitus-associated hearing loss (5.3–5.5).

### 5.1. Genetic and Acquired Hearing Loss

#### 5.1.1. Molecular Therapies

Intensive research is directed toward protecting the sensory hair cells, the auditory neurons and the ribbon synapses connecting the hair cells, focusing primarily on gene and cell-based therapies [38,68]. Goals are to prevent primary auditory neuron loss and support regrowth of auditory neuron fibers in severe hearing loss—both genetic loss and acquired loss such as NIHL. Some of the drugs being tested are being used with cochlear implants and others involve drugs delivered locally to the cochlea.

#### 5.1.2. Stem Cell Therapy

Stem cell differentiation and regeneration of hair cells has been found promising; if regeneration of hair cells were possible, noise-induced hearing losses could be prevented or corrected entirely [18]. Differentiation of stem cells into hair cells and auditory neurons had a high success rate in animal studies, restoring hearing in deafferented animals [69]. Such cell-based therapy may ultimately afford more patients the opportunity to receive cochlear implants.

### 5.2. Tinnitus

#### 5.2.1. Central Auditory Plasticity

Experimental studies targeting central auditory plasticity are one approach. For example, animal studies are targeting maladaptive plasticity in the brain regions of animals with tinnitus, either aiming to reverse the pathological neural activity representing phantom perception of sounds (vagus nerve stimulation (VNS) combined with sound stimulation to alter cortical plasticity) [70], or aiming to reverse tinnitus activity directly, targeting maladaptive plasticity in the fusiform cells in the dorsal cochlear nucleus by using bimodal (auditory-somatosensory) stimulation [71]. Treatments that aim to reverse the pathological neural activity representing phantom perception of sounds are based on neuronal measurements in animal models. Both of these potential treatments are in clinical trials and have emerged from precise single neuron studies, while using GPIAS to assess tinnitus [53]. At present, there are no cures for tinnitus and research interest is especially high.

#### 5.2.2. Neural Plasticity

In a review by Shore et al. [38], altering cortical plasticity to increase the representation of sounds outside the tinnitus region in order to normalize the activity across the primary auditory cortex was able to be achieved by combining VNS with sound stimulation. Another promising method that aims to reverse tinnitus activity directly targets maladaptive plasticity in the fusiform cells in the dorsal cochlear nucleus, which is done using bimodal (auditory-somatosensory) stimulation to induce long-term depression (LTD) in the neurons showing increased long-term potentiation linked to tinnitus [71]. In human subjects, a 28-day LTD-target bimodal stimulation reduced the loudness of tinnitus as well as its intrusiveness.

#### 5.2.3. Modulation of Potassium Channels

Although many pharmacological approaches have been investigated for treating tinnitus, none have produced consistent effects on tinnitus loudness [72]. However, potassium channel modulators (e.g., retigabine, indicated for epilepsy) have been showing promise in animal studies [73], and may have further potential in tinnitus relief by altering potassium currents that cause hyperexcitability in the nervous system. Noise-exposed mice that have ongoing Kv7 activity reduction could develop tinnitus. Mice that reestablish Kv7 activity after noise exposure appear to be resistance to tinnitus [74], suggesting a drug targets for tinnitus pathophysiology.

## 6. Screening and Prevention Strategies

The increasing trends in the incidence and prevalence of hearing loss are due in part to demographic changes such as global population growth, the increased proportion of older adults (>age 65 years) and increased life expectancy [1]. However, while sensorineural hearing loss associated with aging is usually considered the major cause of permanent hearing impairment along with common ear conditions such as chronic otitis media and childhood diseases (e.g., measles, mumps, rubella), NIHL from exposure to occupational and recreational noise is also recognized as a prominent cause—and noise pollution, too, is increasing. Because the incidence of some types of hearing loss could be reduced through prevention, WHO began in 2007 to promote hearing health education and hearing healthcare, to increase public awareness of hearing loss and to develop preventive strategies to reduce exposure to known risk factors such as occupational and recreational noise [6].

As awareness of the adverse effects of noise increases, healthcare providers are more often recommending personal hearing protection (PHP) and hearing protection devices (HPD); only about 8% of surveyed U.S. adults report using HPDs at loud sporting or entertainment events [75]. Simple preventive measures are available to help the millions of individuals affected by excessive noise exposure, not only in terms of hearing loss and tinnitus but of adverse health outcomes such as sleep, blood pressure, cognition, mental health, and quality of life.

Workplace prevention plans for hearing loss are characteristically based on Occupational Safety and Health Act (OSHA)\ regulations that impose strict limits on noise exposure. A recent study exploring whether noise-induced cochlear neuropathy occurring in rodents may justify changes in OSHA regulations argued that (1) humans are less susceptible to temporary threshold shift and cochlear damage than rodents, and (2) exposures that cause cochlear damage in rodents already exceed OSHA limits [76]. Therefore, the authors reasoned that it would be premature to consider that existing noise exposure permissible under OSHA may result in cochlear neuropathy in humans. Various industries in the U.S., including commercial airlines, coal mining, ship building, construction and certain manufacturing processes, as well as military operations, attempt to follow comprehensive OSHA-guided workplace prevention plans to protect employees’ hearing health.

## 7. Conclusions

All people are exposed to noise. However, while the pathogenesis of NIHL is complex, cochlear neuropathy is largely preventable. Advances in our understanding of neural plasticity and adaptive changes in the central auditory system suggest that reversing transient abnormalities when they first emerge may be possible, but ongoing research remains critical, particularly in the assessment and early detection of hearing difficulties before they become permanent. Results of animal and human studies are essential to improve our understanding of neural mechanisms underlying the generation of NIHL, and are critical for developing effective treatment and therapies. Studies must continue to investigate ways to reduce NIHL and improve the quality of life for those who have sustained such hearing impairment. Because no treatment is yet able to completely reverse cochlear damage, continual exploration of new treatments for hearing impairment, implementation of preventative strategies and awareness of hearing health is also crucial.

## References

[1] Olusanya B.O., Davis A.C., Hoffman H.J. (2019). Hearing loss: Rising prevalence and impact. Bull. World Health Organ..

[2] Chadha S., Cieza A. (2017). Promoting global action on hearing loss: World hearing day. Int. J. Audiol..

[3] Kerns E., Masterson E.A., Themann C.L., Calvert G.M. (2018). Cardiovascular conditions, hearing difficulty, and occupational noise exposure within US industries and occupations. Am. J. Ind. Med..

[4] Masterson E.A., Themann C.L., Calvert G.M. (2018). Prevalence of hearing loss among noise-exposed workers within the agriculture, forestry, fishing, and hunting sector, 2003–2012. Am. J. Ind. Med..

[5] Feder K., Michaud D., McNamee J., Fitzpatrick E., Davies H., Leroux T. (2017). Prevalence of Hazardous Occupational Noise Exposure, Hearing Loss, and Hearing Protection Usage Among a Representative Sample of Working Canadians. J. Occup. Environ. Med..

[6] (2018). Addressing the Rising Prevalence of Hearing Loss; World Health Organization: Geneva, Switzerland. http:www.who.int/pbd/deafness/estimates/en.

[7] Lie A., Skogstad M., Johannessen H.A., Tynes T., Mehlum I.S., Nordby K.C., Engdahl B., Tambs K. (2016). Occupational noise exposure and hearing: A systematic review. Int. Arch. Occup. Environ. Health.

[8] Chang S.J., Chang C.K. (2009). Prevalence and risk factors of noise-induced hearing loss among liquefied petroleum gas (LPG) cylinder infusion workers in Taiwan. Ind. Health.

[9] Chang S.J., Chen C.J., Lien C.H., Sung F.C. (2006). Hearing loss in workers exposed to toluene and noise. Environ. Health Perspect..

[10] Chen J.D., Tsai J.Y. (2003). Hearing loss among workers at an oil refinery in Taiwan. Arch. Environ. Health.

[11] Chiu K.W., Lu L.S., Wu C.K. (2015). High pressure air jet in the endoscopic preparation room: Risk of noise exposure on occupational health. Biomed. Res. Int..

[12] Myers J., John A.B., Kimball S., Fruits T. (2016). Prevalence of tinnitus and noise-induced hearing loss in dentists. Noise Health.

[13] Weier M.H. (2020). The Association Between Occupational Exposure to Hand–Arm Vibration and Hearing Loss: A Systematic Literature Review. Saf. Heal. Work..

[14] Government of Canada (2008). Canada Occupational Health and Safety Regulations.

[15] (2006). Industrial Safety and Health Act. Japan. https://www.jisha.or.jp/english/act/index.html.

[16] (1970). Occupational Safety and Health Act of 1970; Public Law 91-596; 1970. Department of Labor, USA. https://www.osha.gov/laws-regs/oshact/toc.

[17] (2019). Occupational Safety and Health Act. 2019 Amendments. Ministry of Labor, Taiwan. https://law.moj.gov.tw/ENG/LawClass/LawAll.aspx?pcode=N0060001.

[18] Imam L., Hannan S.A. (2017). Noise-induced hearing loss: A modern epidemic?. Br. J. Hosp. Med..

[19] Warner-Czyz A.D., Cain S. (2016). Age and gender differences in children and adolescents’ attitudes toward noise. Int. J. Audiol..

[20] Gong R., Hu X., Gong C., Long M., Han R., Zhou L., Wang F., Zheng X. (2018). Hearing loss prevalence and risk factors among older adults in China. Int. J. Audiol..

[21] Shore S.E., Roberts L.E., Langguth B. (2016). Maladaptive plasticity in tinnitus—Triggers, mechanisms and treatment. Nat. Rev. Neurol..

[22] Vanneste S., De Ridder D. (2012). The auditory and non-auditory brain areas involved in tinnitus. An emergent property of multiple parallel overlapping subnetworks. Front. Syst. Neurosci..

[23] Rauschecker J.P., Leaver A.M., Muhlau M. (2010). Tuning out the noise: Limbic-auditory interactions in tinnitus. Neuron.

[24] Leaver A.M., Turesky T.K., Seydell-Greenwald A., Morgan S., Kim H.J., Rauschecker J.P. (2016). Intrinsic network activity in tinnitus investigated using functional MRI. Hum. Brain Mapp..

[25] Kujawa S.G., Liberman M.C. (2015). Synaptopathy in the noise-exposed and aging cochlea: Primary neural degeneration in acquired sensorineural hearing loss. Hear. Res..

[26] Roberts L.E., Eggermont J.J., Caspary D.M., Shore S.E., Melcher J.R., Kaltenbach J.A. (2010). Ringing ears: The neuroscience of tinnitus. J. Neurosci..

[27] Landgrebe M., Zeman F., Koller M., Eberl Y., Mohr M., Reiter J., Staudinger S., Hajak G., Langguth B. (2010). The Tinnitus Research Initiative (TRI) database: A new approach for delineation of tinnitus subtypes and generation of predictors for treatment outcome. BMC Med. Inform. Decis Mak..

[28] Weisz N., Moratti S., Meinzer M., Dohrmann K., Elbert T. (2005). Tinnitus perception and distress is related to abnormal spontaneous brain activity as measured by magnetoencephalography. PLoS Med..

[29] Lanting C.P., de Kleine E., van Dijk P. (2009). Neural activity underlying tinnitus generation: Results from PET and fMRI. Hear. Res..

[30] Dos Santos Filha V.A., Samelli A.G., Matas C.G. (2015). Middle Latency Auditory Evoked Potential (MLAEP) in Workers with and without Tinnitus who are Exposed to Occupational Noise. Med. Sci. Monit..

[31] Lee C.F., Lin M.C., Lin H.T., Lin C.L., Wang T.C., Kao C.H. (2016). Increased risk of tinnitus in patients with temporomandibular disorder: A retrospective population-based cohort study. Eur. Arch. Otorhinolaryngol..

[32] Wang T.C., Tyler R.S., Chang T.Y., Chen J.C., Lin C.D., Chung H.K., Tsou Y.A. (2018). Effect of Transcranial Direct Current Stimulation in Patients With Tinnitus: A Meta-Analysis and Systematic Review. Ann. Otol. Rhinol. Laryngol..

[33] Tyler R.S., Aran J.M., Dauman R. (1992). Recent Advances in Tinnitus. Am. J. Audiol..

[34] Salvi R., Boettcher F.A. (2008). Animal Models of Noise-Induced Hearing Loss. Sourcebook of Models for Biomedical Research.

[35] Shulman A., Goldstein B., Strashun A.M. (2009). Final common pathway for tinnitus: Theoretical and clinical implications of neuroanatomical substrates. Int. Tinnitus J..

[36] Ding T., Yan A., Liu K. (2019). What is noise-induced hearing loss?. Br. J. Hosp. Med..

[37] Martin W.H., Sobel J., Griest S.E., Howarth L., Yongbing S.H.I. (2006). Noise induced hearing loss in children: Preventing the silent epidemic. J. Otol..

[38] Shore S.E., Wu C. (2019). Mechanisms of Noise-Induced Tinnitus: Insights from Cellular Studies. Neuron.

[39] Ralli M., Gilardi A., Stadio A.D., Severini C., Salzano F.A., Greco A., de Vincentiis M. (2019). Hearing loss and Alzheimer’s disease: A review. Int. Tinnitus J..

[40] Walker J.J., Cleveland L.M., Davis J.L., Seales J.S. (2013). Audiometry screening and interpretation. Am. Fam. Physician.

[41] Cueva R.A. (2004). Auditory brainstem response versus magnetic resonance imaging for the evaluation of asymmetric sensorineural hearing loss. Laryngoscope.

[42] Ratnanather J.T. (2020). Structural neuroimaging of the altered brain stemming from pediatric and adolescent hearing loss-Scientific and clinical challenges. Wiley Interdiscip. Rev. Syst. Biol. Med..

[43] Vos S.B., Haakma W., Versnel H., Froeling M., Speleman L., Dik P., Viergever M.A., Leemans A., Grolman W. (2015). Diffusion tensor imaging of the auditory nerve in patients with long-term single-sided deafness. Hear. Res..

[44] Lin Y., Wang J., Wu C., Wai Y., Yu J., Ng S. (2008). Diffusion tensor imaging of the auditory pathway in sensorineural hearing loss: Changes in radial diffusivity and diffusion anisotropy. J. Magn. Reson. Imaging.

[45] Zhang K.D., Coate T.M. (2017). Recent advances in the development and function of type II spiral ganglion neurons in the mammalian inner ear. Semin. Cell Dev. Biol..

[46] Grandi F.C., De Tomasi L., Mustapha M. (2020). Single-Cell RNA Analysis of Type I Spiral Ganglion Neurons Reveals a Lmx1a Population in the Cochlea. Front. Mol. Neurosci..

[47] Tyler R.S., Conrad-Armes D. (1983). The determination of tinnitus loudness considering the effects of recruitment. J. Speech Hear. Res..

[48] Tyler R.S., Oleson J., Noble W., Coelho C., Ji H. (2007). Clinical trials for tinnitus: Study populations, designs, measurement variables, and data analysis. Prog. Brain Res..

[49] Tyler R.S., Tyler R.S. (2000). The psychoacoustical measurement of tinnitus. Tinnitus Handbook.

[50] Vernon J.A., Meikle M.B. (2003). Tinnitus: Clinical measurement. Otolaryngol. Clin. N. Am..

[51] Tyler R., Ji H., Perreau A., Witt S., Noble W., Coelho C. (2014). Development and validation of the tinnitus primary function questionnaire. Am. J. Audiol..

[52] Bakay W.M.H., Anderson L.A., Garcia-Lazaro J.A., McAlpine D., Schaette R. (2018). Hidden hearing loss selectively impairs neural adaptation to loud sound environments. Nat. Commun..

[53] Galazyuk A., Hebert S. (2015). Gap-Prepulse Inhibition of the Acoustic Startle Reflex (GPIAS) for Tinnitus Assessment: Current Status and Future Directions. Front. Neurol..

[54] Lambriks L.J.G., van Hoof M., Debruyne J.A., Janssen M., Chalupper J., van der Heijden K.A., Hof J.R., Hellingman C.A., George E.L.J., Devocht E.M.J. (2020). Evaluating hearing performance with cochlear implants within the same patient using daily randomization and imaging-based fitting—The ELEPHANT study. Trials.

[55] Sanderson A.P., Rogers E.T.F., Verschuur C.A., Newman T.A. (2018). Exploiting Routine Clinical Measures to Inform Strategies for Better Hearing Performance in Cochlear Implant Users. Front. Neurosci..

[56] Nishad R.K., Jain A.K., Singh M., Verma R., Jain S. (2019). Randomised Controlled Clinical Study of Injection Caroverine and Ginkgo Biloba Extract in Cochlear Synaptic Tinnitus. Indian J. Otolaryngol. Head Neck Surg..

[57] Elzayat S., Ragab S., Eisa M., Amer M., Mandour M.F., Mehraz M. (2018). Evaluation of Adding Lidocaine to Dexamethasone in the Intra-tympanic Injection for Management of Tinnitus: A Prospective, Randomized, Controlled Double-blinded Trial. Int. Tinnitus J..

[58] Kumral T.L., Yildirim G., Berkiten G., Salturk Z., Atac E., Atar Y., Uyar Y. (2016). Efficacy of Trimetazidine Dihydrochloride for Relieving Chronic Tinnitus: A Randomized Double-Blind Study. Clin. Exp. Otorhinolaryngol..

[59] Honkura Y., Matsuo H., Murakami S., Sakiyama M., Mizutari K., Shiotani A., Yamamoto M., Morita I., Shinomiya N., Kawase T. (2016). NRF2 Is a Key Target for Prevention of Noise-Induced Hearing Loss by Reducing Oxidative Damage of Cochlea. Sci. Rep..

[60] Bentler R.A., Tyler R.S. (1987). Tinnitus management. ASHA.

[61] Jastreboff P.J. (2015). 25 years of tinnitus retraining therapy. HNO.

[62] Tunkel D.E., Bauer C.A., Sun G.H., Rosenfeld R.M., Chandrasekhar S.S., Cunningham E.R., Archer S.M., Blakley B.W., Carter J.M., Granieri E.C. (2014). Clinical practice guideline: Tinnitus. Otolaryngol. Head Neck Surg..

[63] Kochkin S., Tyler R.S. (2008). Tinnitus Treatment and the Effectiveness of Hearing Aids: Hearing Care Professional Perceptions. Hear. Rev..

[64] Tyler R.S., Perreau A., Powers T., Watts A., Owen R., Ji H., Mancini P.C. (2020). Tinnitus Sound Therapy Trial Shows Effectiveness for Those with Tinnitus. J. Am. Acad. Audiol..

[65] Rojas-Roncancio E., Tyler R., Jun H.J., Wang T.C., Ji H., Coelho C., Witt S., Hansen M.R., Gantz B.J. (2016). Manganese and Lipoflavonoid Plus((R)) to Treat Tinnitus: A Randomized Controlled Trial. J. Am. Acad. Audiol..

[66] Coelho C., Tyler R., Ji H., Rojas-Roncancio E., Witt S., Tao P., Jun H.J., Wang T.C., Hansen M.R., Gantz B.J. (2016). Survey on the Effectiveness of Dietary Supplements to Treat Tinnitus. Am. J. Audiol..

[67] Tyler R., Coelho C., Tao P., Ji H., Noble W., Gehringer A., Gogel S. (2008). Identifying tinnitus subgroups with cluster analysis. Am. J. Audiol..

[68] Ma Y., Wise A.K., Shepherd R.K., Richardson R.T. (2019). New molecular therapies for the treatment of hearing loss. Pharmacol. Ther..

[69] Dufner-Almeida L.G., da Cruz D.B., Mingroni Netto R.C., Batissoco A.C., Oiticica J., Salazar-Silva R. (2019). Stem-cell therapy for hearing loss: Are we there yet?. Braz. J. Otorhinolaryngol..

[70] Engineer C.T., Engineer N.D., Riley J.R., Seale J.D., Kilgard M.P. (2015). Pairing Speech Sounds With Vagus Nerve Stimulation Drives Stimulus-specific Cortical Plasticity. Brain Stimul..

[71] Marks K.L., Martel D.T., Wu C., Basura G.J., Roberts L.E., Schvartz-Leyzac K.C., Shore S.E. (2018). Auditory-somatosensory bimodal stimulation desynchronizes brain circuitry to reduce tinnitus in guinea pigs and humans. Sci. Transl. Med..

[72] Langguth B., Elgoyhen A.B. (2012). Current pharmacological treatments for tinnitus. Expert Opin. Pharmacother..

[73] Jentsch T.J. (2000). Neuronal KCNQ potassium channels: Physiology and role in disease. Nat. Rev. Neurosci..

[74] Li S., Choi V., Tzounopoulos T. (2013). Pathogenic plasticity of Kv7.2/3 channel activity is essential for the induction of tinnitus. Proc. Natl. Acad. Sci. USA.

[75] Eichwald J., Scinicariello F., Telfer J.L., Carroll Y.I. (2018). Use of personal hearing protection at athletic or entertainment events among adults—United States, 2018. CDC Wkly..

[76] Dobie R.A., Humes L.E. (2017). Commentary on the regulatory implications of noise-induced cochlear neuropathy. Int. J. Audiol..

